# Characteristics and Validity of a Web-Based Kawasaki Disease Surveillance System in Japan

**DOI:** 10.2188/jea.JE20100006

**Published:** 2010-11-05

**Authors:** Yosikazu Nakamura, Mayumi Yashiro, Ryusuke Ae, Izumi Chihara, Atsuko Sadakane, Yasuko Aoyama, Kazuhiko Kotani, Ritei Uehara, Shohei Harada

**Affiliations:** 1 Department of Public Health, Jichi Medical University, Shimotsuke, Tochigi, Japan; 2 Department of Health Policy, National Research Institute for Child Health and Development, National Center for Child Health and Development, Tokyo, Japan

**Keywords:** mucocutaneous lymph node syndrome, incidence, epidemiology, sentinel surveillance, internet

## Abstract

**Background:**

Although regular nationwide surveys of Kawasaki disease (KD) are conducted in Japan, there is no system for detecting the real-time epidemic status of this disease.

**Methods:**

A web-based surveillance system for KD was developed. After consideration of the number of patients reported by prefecture to the 19th nationwide survey, 355 pediatric departments were asked to participate in the surveillance, and 225 agreed. Since January 2008, pediatricians in these 225 hospitals have reported KD patient data immediately after diagnosis. The daily numbers of patients are available to the public via the internet at http://www.kawasaki-disease.net/kawasakidata/. The validity of the data in 2008 was evaluated using the Japanese 20th nationwide survey of KD as the gold standard.

**Results:**

A total of 3376 patients were reported to the web-based surveillance system from the 1st week through 52nd week of 2008. The number of patients reported to the nationwide survey during the same period was 11 680: a total of 4950 patients from the hospitals participating in the web-based surveillance and 6730 from other hospitals. The epidemic curves were similar, and the correlation coefficient between the web-based surveillance and the total numbers in the nationwide survey was 0.806 (*P* < 0.01).

**Conclusions:**

The web-based surveillance system for Kawasaki disease in Japan demonstrated good validity.

## INTRODUCTION

Kawasaki disease (KD) affects mainly infants and toddlers. The number of patients with KD and its incidence rate have increased year by year in Japan, and the total number of patients who have received a diagnosis of KD in Japan is 249 019.^1^ However, the etiology of the disease remains unknown. Nationwide epidemiologic surveys are conducted every 2 years to observe the epidemiologic features of the disease in Japan.^1^^,^^2^ Many epidemiologic and clinical features have been revealed by analyzing the data from these surveys, but the surveys have some limitations. One of the most important of these is time lag. Because the survey is biennial, we do not obtain information on the real-time frequency of KD.

To solve this problem, a research committee established a KD surveillance system and asked pediatricians to use a postcard to continuously report the monthly number of patients.^3^^–^^5^ This committee-based monthly surveillance ended in 1996, after KD was designated as a target infectious disease. As a result of this new designation, KD became part of a weekly national surveillance system for infectious diseases conducted by the Japanese Ministry of Health and Welfare (currently the Ministry of Health, Labour and Welfare). The validity of this national surveillance system was confirmed in a comparison with data from the nationwide surveys.^6^ However, this national surveillance system was changed in 1999, after the Infectious Disease Prevention Act of 1900 was superceded by the current Prevention of Infectious Diseases and Medical Care for Patients Suffering Infectious Diseases Act of 1998, which excluded KD from the list of target diseases subject to national surveillance.^6^ As a result, there was no real-time surveillance of KD in Japan from 1999 until 2007.

In 2007, the Research Committee on Study on the Construction of Comprehensive Data Base about the Chronic Diseases of Children (Chairman: Dr. Shohei Harada) established a new web-based surveillance system for KD in Japan. The system started in October 2007, and a fully operational system has been available since January 2008. In this report, we explain the web-based surveillance system and evaluate its validity using data from the 20th nationwide survey of KD.

## METHODS

The web-based surveillance system for KD was constructed by a communication company (Ohmi Computer System, Ltd.). Immediately after a diagnosis of KD is made by a pediatrician, patient data are entered, including patient name (initials only), sex, address (municipality name only), date of birth, date of onset, date of first visit to the medical institution, date of diagnosis, and diagnosis (typical definite: 5 or 6 of the principal symptoms, according to the diagnostic guidelines of the disease^7^; atypical definite: 4 principal symptoms plus cardiac lesion[s]; suspected type A: 4 principal symptoms without cardiac lesions; and suspected type B: 3 or fewer principal symptoms and cardiac lesion[s]). The registered data are entered on a personal computer by the pediatrician, encrypted, sent to a server of the communication company, and stored using a secure system requiring passwords. The daily numbers of patients registered can be seen in real-time by the public via the internet (http://www.kawasaki-disease.net/kawasakidata/). Information on the age and sex distribution of all registered patients is also available. In addition, a pediatrician can analyze patient data that he/she has registered, and participating hospitals can observe the epidemic curve by district.

In 2007, we recruited hospitals from which pediatricians would enter patient data into the system. Using data from the 19th nationwide survey of KD,^2^ we identified the hospitals that reported the 3 highest numbers of patients in each prefecture and asked them to participate in the web-based surveillance; any additional hospitals with 26 or more patients during the period from 2005 through 2006 (the target years of the 19th survey) were also asked to participate. Ultimately, 355 hospitals were asked to participate in the web-based surveillance system, and 225 eventually did so. Pediatricians in these 225 hospitals have entered the required patient data since October 2007.

The validity of the web-based surveillance system was evaluated using the 20th nationwide survey of KD^1^ as the gold standard. The weekly numbers of patients (from Monday through Sunday) who first visited a hospital because of KD in 2008 were calculated using data from the web-based surveillance system and the 20th nationwide survey. In detail, the weekly numbers of patients for the 2 surveys were compared from the 1st week of 2008, which started on 31 December 2007, through the 52nd week, which ended on 28 December 2008. In addition to the overall analysis, data from the nationwide survey were classified by whether the hospital had participated in the web-based surveillance system or not. The correlation coefficients (degrees of freedom = 50) between the weekly numbers of patients reported to the web-based surveillance and nationwide survey were calculated.

The Ethical Committee on Epidemiologic Research of Jichi Medical University approved the study (13 September 2007, Eki 07-17).

## RESULTS

**Figure 1.**
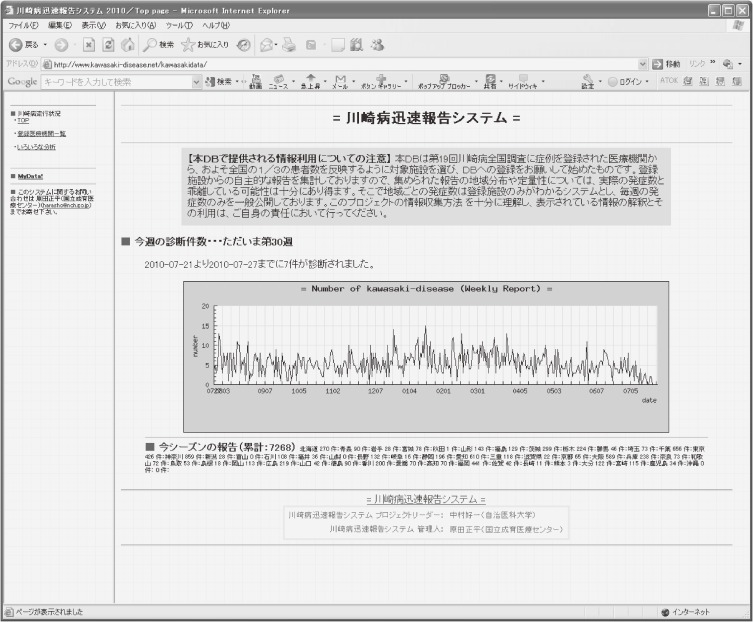
System used for surveillance of Kawasaki disease (screenshot) http://www.kawasaki-disease.net/kawasakidata/

The web-based surveillance system has been fully operational since January 2008, and 5837 patients were reported by the end of 2009 (http://www.kawasaki-disease.net/kawasakidata/). In January 2009, 10 to 20 new patients were reported to the system each day. The daily number of new patients subsequently declined to 5 to 10, as shown in Figure 1. Since July 2009, the daily number was approximately 5. The number of male patients was 3325, and the number of females was 2480 (the sex of 32 patients is unknown). The age distribution peaked at 6 to 11 months. These findings were similar to the epidemiologic features reported in the nationwide surveys.^1^^,^^2^

**Figure 2.**
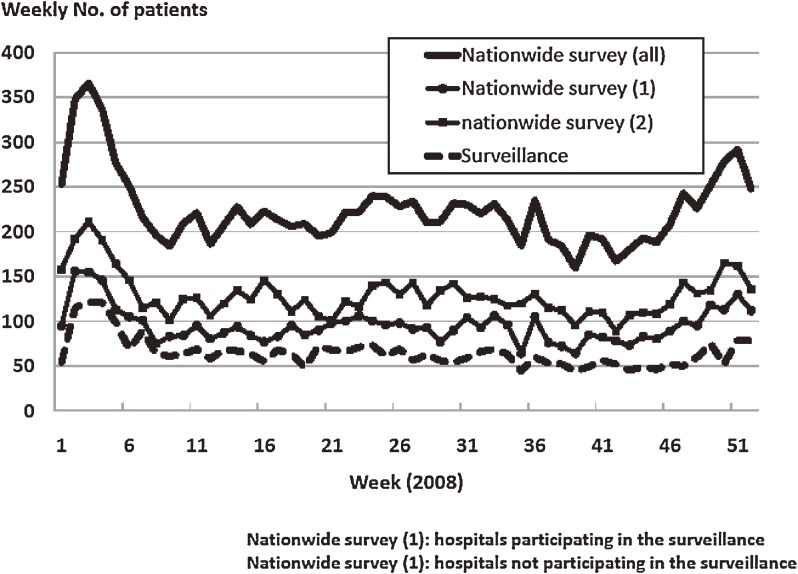
Weekly numbers of patients with Kawasaki disease reported to the internet surveillance system and the 20th nationwide survey in 2008.

A total of 3376 patients were reported by the 225 hospitals participating in the web-based surveillance system from the 1st through the 52nd week of 2008. The number reported to the nationwide survey was 11 680 (4950 patients from the 225 hospitals participating in the web-based surveillance and 6730 from other hospitals). Figure 2 shows the weekly numbers of patients from the 2 data sources. The epidemic curves were similar, and the correlation coefficient was 0.806 for the total numbers of patients reported to the web-based system and the 20th nationwide survey, 0.852 for the web-based surveillance data and data from the 20th nationwide survey reported by hospitals participating in the web-based surveillance, and 0.694 for the web-based surveillance data and data from the 20th nationwide survey reported by other hospitals. All the coefficients were significant (*P* < 0.01).

## DISCUSSION

In this report, we described the current state of a web-based surveillance system for KD and noted its high validity in comparison with data from the 20th nationwide survey of the disease. Because as many as 225 hospitals have participated in the web-based surveillance, the data collected are valid, and we are able to observe the real-time epidemic curve of KD in Japan.

Although there have been approximately 250 000 patients with KD in Japan, the etiology of the disease remains unknown. However, epidemiologic data indicate that an infectious agent or agents is related to the onset of KD, as there have been 3 nationwide epidemics, in 1979, 1982, and 1986,^1^^,^^2^ and investigators have noted a monomodal age distribution,^1^^,^^2^ time and geographical clustering of the patients,^8^ and a high frequency of the disease among siblings of KD patients.^8^ In addition, nationwide survey data^8^ revealed seasonal incidence, which has also been noted in other countries.^9^ Thus, we believe that it is important to monitor the number of patients with KD, as is done with infectious diseases, even though KD is not certified as infectious.

There have been several validation studies of KD surveillance systems in Japan, all of which used nationwide survey data as the gold standard.^6^^,^^10^^,^^11^ In this study we also used these data as the gold standard.

Many hospitals joined the web-based surveillance system because of 2 advantages: they are able to use their the own data in the system and they can observe detailed analyses. The former means that a pediatrician can use his/her registering data via the internet, and the latter allows observation of epidemic curves by district. These 2 advantages are incentives for participation.

Of the 11 680 KD patients reported to the nationwide survey for 2008, 3376 were posted to the web-based surveillance system as well, a proportion of 28.9%. In the previous postcard surveillance system administered by the research committee, the reported number of patients was approximately one-third that reported to the nationwide survey for the corresponding time period.^3^ Because the previous system was able to detect the third nationwide epidemic of the disease, in 1986,^3^^,^^5^ we believe that the size of the current web-based surveillance system is sufficient for detecting epidemics, as its sample size is similar to that of the previous system.

The large number of participating hospitals ensured that the data of the web-based surveillance system had high validity, as determined using the nationwide survey as gold standard. Correlation coefficients are the main index of the external validity for continuous data,^12^ and all were close to 1.0 in the present study. The epidemic curve of patients from hospitals not participating in the web-based surveillance was fairly similar to that of patients from participating hospitals. These results imply that the number of patients from non-participating hospitals increased when the total number of patients increased, because the numbers of patients with KD per hospital were somewhat smaller than those in participating hospitals. This indicates that patients with KD visited hospitals without consideration of the number of patients visiting the hospitals. Therefore, the epidemic curve based on the surveillance data resembled the gold standard, ie, the epidemic curve from the nationwide survey data.

In conclusion, the current web-based surveillance of KD demonstrated good validity.
